# Modelling the inhibitors of cold supply chain using fuzzy interpretive structural modeling and fuzzy MICMAC analysis

**DOI:** 10.1371/journal.pone.0249046

**Published:** 2021-04-13

**Authors:** Anshuman Sharma, Haidar Abbas, Muhammad Qutubuddin Siddiqui

**Affiliations:** 1 College of Business Administration, Ajman University, Ajman, United Arab Emirates; 2 Salalah College of Applied Sciences, University of Technology and Applied Sciences, Salalah, Oman; University of Defence in Belgrade, SERBIA

## Abstract

The Cold Supply Chain (CSC) is an integral part of the supply chain of perishable products. The aim of this research is to examine the inhibitors that have a major impact on the performance of CSC operations in the United Arab Emirates (UAE). This study provides a synthesis and suggests a hierarchical model among CSC inhibitors and their respective relevance. The hierarchical synthesis of twelve (12) primary CSC inhibitors is achieved through a comprehensive literature review and consultation with academics and CSC professionals. This study used semi-structured interviews, a fuzzy interpretive structural modeling (FISM) and a Fuzzy-MICMAC (FMICMAC) analysis to explore and establish the relationship between and among identified inhibitors. FISM is used to examine the interaction between inhibitors, while FMICMAC analysis is used to examine the nature of inhibitors on the basis of their dependence and driving power. The results of the FISM and FMICMAC analysis show the inter-relationships and relative dominance of identified inhibitors. The results show that some inhibitors are of high strategic importance due to their high driving power and low dependence. These inhibitors seek more management attention in order to improve their effectiveness. The result of a hierarchical model helps to understand the influence of a particular inhibitor on others. ‘Higher capital and operating costs’ occupy the highest level in the FISM model. The ‘fragmented cold supply chains’, ‘lack of skilled labor’, ‘inadequate information system infrastructure’ and ‘lack of commitment by top level management’ had strong driving power but weak dependence, which characterizes them as independent inhibitors. Management should be extra careful when dealing with these inhibitors as they influence the effects of other variables at the top of the FISM hierarchy in the overall management of the cold supply chain. The study also suggests a number of recommendations for addressing these inhibitors in cold supply chains operating in the UAE. With due attention and care for these inhibitors, the operation of the cold supply chains is likely to be even more successful.

## Introduction & review of previous studies

On account of changing preferences owing to lifestyle in metro cities, high-income levels, rising middle class, and growing urbanization [1], there has been an ever-increasing dependency on organized retail stores along with round-the corner fast-food chains in the UAE. McCrory et al. [2] mention that fast food restaurants around the world are on the rise. The cold storage chains are thereby the life-blood for frozen (food) businesses. However, there are many quality and safety issues associated with ‘frozen food’ [3]. A recent positive drift of research studies in this area is also presumed to have contributed to the emergence of cold supply chain management as an adjunct discipline of knowledge [4]. For example, the pressure for sustainable supply chains as delineated by the triple bottom line (economic, social and environmental) [5] has received increased attention from researchers. Furthermore, the stringent government regulations and growing public awareness about environmental issues have convinced many firms to incorporate the green perspective into their supply chains [6]. Despite all, there remains a paucity of research in this field [7]. The established research agenda based on a systematic literature review placed identification of the factors that adversely influence the cold food chain management [8].

Perishability is one of the most significant aspects, which distinguishes the temperature-sensitive (cold) supply chains from the rest of the supply chains. These supply chains reflect a blend of organization, sharing, and integration of relevant information [9]. It is the temperature, which essentially determines the shelf life of perishable products [10] like milk and milk related products, meat, poultry products, fish, fruits, vegetables, etc. In fact, for a few products, humidity is also essential [11]. One must note here that storing such products at consistently recommended conditions is critical for their utility. Storing is done by keeping them intact using pallets, crates, cartons, glass/plastic bottles, and other suitable containers. This, in turn, improves their shelf life and ensures that their quality is maintained over an extended shipping period, while minimizing the chances of waste, waste disposal costs, and associated emissions.

Due to the involvement of multiple handlers, coupled with long-distance travel, managing the variability of temperature and other recommended conditions during transit and transfers is one of the many unique challenges in the industry; add to that poor circulation of air, radiant heat from the floor, substandard insulated areas, and/or refrigerant leakages, and you have an unhealthy environment. It is important to note that in case of any regulatory infringement on maintaining the recommended conditions during transit, it is actually difficult to hold a specific person or an entity accountable, as this supply chain comprises of multiple players; potentially, each one of them could contribute to the loss/degradation of product quality. In essence, it is the lack of visibility [12] which is the primary inhibitor in such cases.

Real-time condition (temperature/humidity) monitoring technology [13], the declining cost of internet of things (IoT) technology [4], and analysis enabled sensors like thermal imaging and refrigeration leak detection already exist across the value chains, particularly in the food and the pharmaceutical industries. Typical constraints that were faced earlier relating to IoT, like the packet size, speed of network packets, and packet loss [14] have also been addressed to a great extent thus far; however, the network disruption, due to both controllable and uncontrollable factors, still exists.

Cold chain logistics, especially in the food-processing sector, being at a nascent stage in most of the developing countries [15], include inhibitors such as the non-availability of critical infrastructure facilities like packing, quality testing centers [3], cold storage facilities, and weak links of the fruit and vegetable cold chain [16]. Add to this the lack of upgraded and advanced technology, which becomes one of the biggest inhibitors to the success of the food processing industry, which in turn affects the cold chain management [17]. Effective integration of the cold storage supply chain sector requires state of the art static infrastructure, mobile infrastructure, standards and protocols, and skilled resources.

Besides enhancing customer satisfaction [18], improved tracking through IoT based cargo management system may address many of the reported cold supply chain issues. It may provide benefits like improved product value (due to extended shelf life, de- creased product loss / decreased discount due to improved quality) reduced indirect logistics costs, reputational benefits (for being more consistent) and reduced carbon emissions due to controlled energy usage related to refrigerant leakage and emissions that result in food waste [19]. In fact, efforts have been made to assess the viability of joint emission reduction efforts in compliance with environmental regulations by a local supply chain involving a manufacturer and a retailer [20]. Furthermore, the likely energy savings would include reduced food waste and reduced loss of operating energy (cooling energy lost from truck doors left open, excessive transfer times and unloading delays) etc.

Nevertheless, there are many challenges associated here, too, while one seeks to gain maximum advantages by implementing such monitoring and reporting systems. Some of the key challenges (inhibitors) include the lack of encouraging evidence, post-harvest losses [21], strict allocation of cost-benefit accountability due to the highly fragmented industry, and a large number of players that already exist in this supply chain: foods; illegal, unreported, and unregulated (IUU) products; higher costs of advanced temperature monitoring automated response systems like RFID instead of simple bar code system [22]; required specialized training [13] and culture change; and costs associated with mandatory refrigerant changeover with upgrading efficiency and flexible capacity [23]. To add to this growing list, the food products being necessarily halal [24], like in the case of Malaysia, adds more to the cold supply chain management issues in the UAE.

The development of a cold supply chain sector is largely determined by uninterrupted supply, and recorded and demonstrated traceability of products [25]. Additionally, customers today are expecting better product quality, higher service levels and lower prices. Inadequate supply chain management skills and qualifications, poor procurement practices, ineffective integration of supply chains, poor supply chain relations and industry structure are considered to be some of the major impediments in supply chains in developing countries. However, supply chain management issues should be considered contextually. Based on systematic analysis of 100 peer-reviewed journal articles on FSCM Scientific databases, Mahajan et al. [26] found that relatively limited research exists in the domain of food supply chain from an empirical prescriptive. Gligor et al., [27] conducted a similar study in the context of Vietnam using a qualitative methodology and suggested that future researchers extend it in the context of another developing country. Within the cold supply chains, there exist numerous disparities. For instance, cold supply chains responsible for managing the pharmaceuticals manufacturing, storage, movement, delivery and return may differ from those responsible for the storage and movement of biological consignments like human organs. Likewise, cold supply chains responsible for managing the flow of human organs may differ in many other aspects from those responsible for flowers. Similarly, cold supply chains ensuring the seamless movement of flowers may differ in certain other aspects from those responsible for foods. One type of these cold supply chains may require the liquid nitrogen or dry ice, whereas, the other one may need gel packs or eutectic plates. Thus, the challenges faced by each of these cold supply chains may significantly differ. Food forms the most important basic need for the existence of human beings. Even those who can’t afford medicines, or organ transplant, they also need some food to survive. Countries and geographical regions differ in terms of arable land, climatic condition, required infrastructure, accessibility etc. and thus, some of them are self-sufficient while some others are partially or completely dependent on others. As per the estimates of Food and Agriculture Organizations of the United Nations, the world hunger is surging on one hand while approximately one-third of the global food production is lost or wasted on the other. All of these issues primarily convinced the researchers to investigate the cold supply chains managing the basic food products and explore various inhibitors encountered by the food processing industry. The identification, examination and establishment of the potential cold supply chain inhibitors may be helpful in comprehending the hunger-food wastage paradox. The subsequent paragraphs underline the motivations behind the selection of UAE as the studied region.

### Scenario of Gulf Cooperation Council (GCC)

Approximately 2% of the total land area in the GCC is being used for agriculture, which contributes to about 1.5% of the GDP. It was estimated that the GCC food import budget, in comparison to 2004, wherein it was valued at $25.8 billion, would surpass $53.1 billion by 2020; this would be equivalent to around 90% of the total food consumption within the region. Being dependent on the Suez Canal and the Straits of Hormuz to procure a considerable quantity of its food, the region is exposed to the geopolitical risk that effectively jeopardizes the food supply chains.

In general, the consumers’ ever increasing demand for organic, more nutritious and low-calorie food across the globe has put forth a different set of challenges for new product development in the food supply chains [28]. However, Ng et al., [29] reported that among UAE nationals, a different nutritional transition like the consumption of sugared sodas, fruit drinks, and whole milk, etc. has posed potential risks for severe cardio-metabolic problems. Haroun et al., [14] found that non-Emirati students eat healthier food than their Emirati counterparts do; however, Emiratis spend more than non-Emiratis.

The Arab World records a per person food wastage that sometimes exceeds 210 kg per year [30]. As the UAE imports approximately 90% of its food, and serves a $9.4 billion market of GCC and Africa, food wastage becomes an issue of burning importance. Among many, the GCC Retail Industry Report [31] acknowledged the dependence on the imported food as one of the major challenges encountered by the GCC’s retail sector. Use of third-party logistics (3PL’s), government regulations, halal certification [32], lean quality management, upstream and downstream collaboration, demand forecasting, customer experience as a source of competitive advantage, would undoubtedly be the need of the hour.

The unique socio-demographic and cultural blend limited by the local agrarian resource base make the nation (UAE) a consumer society, the one that largely hinges on the other agrarian societies in the nearby region. Being a global tourism and trade hub, it attracts the best brains from around the globe to serve across the sectors, particularly the service sector. This culturally diversified customer base offers a plethora of opportunities for the cold supply chains to grow up to their varied expectations in terms of quality, availability, food choices, competitive prices, service delivery etc. Hence, it convinced the researchers to examine how these various aspects register a synergetic effect on the practices of cold supply chains operating in United Arab Emirates. More specifically, the researchers got interested in learning the key challenges that the cold supply chains operating in this region face and how these challenges relate with and contribute to each other to the disadvantage of the cold supply chain operations. The absence of any such structured study in the context of UAE except Tamimi et al [33] makes this study a trailblazer attempt. The outcomes are expected to be equally important to all gulf cooperation council (GCC) countries, which share similar geographic, socio-demographic and natural resource profile.

## Inhibitors to cold supply chain operations

This section briefly explains the selected inhibitors with the help of key relevant and recent studies.

### Fragmented cold supply chains

It involves many business partners, including intermediaries, agents, etc. from source (i.e. farm to fork) to destinations [34]. Farmers generally tend to have small and scattered landholdings [25]. As per the estimates [23], the intricate process generally leads the average food to move more than ten times in and out of refrigeration control system across the entire cold supply chain before reaching the consumers’ fork. Therefore, it is unclear when exactly and where unerringly there possibly would be an abuse in the temperature. As a result, setting accountability in case of any lapses becomes complex. Packaging, being an essential aspect, shields the products from microbiological and sensory deterioration. Thus, the manufacturers and developers in the cold supply chains [35] must respect the experiences of consumers, their requirements, and packaging standards. Oliva and Revetria, [36] identified cross docking, consolidation and a credible temperature assessment method as three interrelated key issues in cold chain management on fresh food.

### Lack of top-level management’s commitment

This refers to the degree of reluctance Vis a Vis strategic viability and operational sustainability of cold supply chain operations, which is exhibited in the attitude of the top-level management. Unavailability of cold chain performance measurements, lack of specific environmental goals, uneven installation of cold chain centers, along with poor collaborative practices reflect the same. Unavailability of lax performance measures affect the risk management mechanism. Attitude towards risk management [37], lack of encouraging evidences [21] and strict allocation of cost-benefit accountability due to the highly fragmented industry coupled with the large number of players could also be some additional factors reasons behind it.

### Poor cold supply chain network

A cold supply chain refers to a network of interdependent enterprises that work together to deliver the best possible customer value (quality consumables) at the minimum possible costs. Due to lack of standardization [34], and improper collaboration among these enterprises, many discrepancies may crop up at different levels, like misleading forecasts, unexpected low/high inventories, labor scheduling issues, or delivery related challenges. Among many, one possible solution could be to integrate the otherwise fragmented players–like seed companies, cooperative societies, farmers, 3 PL service providers, academic and vocational institutions, product value-added producers and, eventually, consumers [38]. An astute integration would result in a robust and resilient supply chain network.

### Poor collaboration

Cold supply chain integrity must be ensured from the point of origin (production), through processing, transits, and delivery for consumption or storage [39]. Farooq et al., [40] acknowledged that the contamination caused by food degradation, food adulteration, counterfeiting or fake labelling etc. are some existing challenges within the industry that actually go on to affect food safety and quality [41], and thereby the consumer’s health and safety. Variation in the regulatory enforcement across the globe further makes the issue more complicated. Lack of certainty or the incapability to coordinate a number of activities with channel members pose several challenges [42], including the feeble trust and collaboration at the buyer-supplier interface, languishing interpersonal relationships, along with the lack of cold supply chain integration further aggravate the situation. Freiboth et al., [43] thereby highlighted the need to improve cold supply chain operational procedures.

### Negligible local production of selected CSC items

Being located in an arid zone (MOCCAE, p-15) [44], characterized with more than 75% desert environment, UAE experiences low rainfall, high temperature, poor soil, and deficient natural waterways. Resultantly, the country’s agricultural and farming sector contributes a negligible amount to the country’s GDP; in fact, experts also categorized this as being a primary inhibitor to be included for further studies. In other words, this situation makes the cold supply chain networks operating in UAE to necessarily have certain stakeholders from outside, over which they may have a limited control.

### Higher capital & operating costs

Fixed costs of installation along with operational costs of the requisite infrastructure are exorbitantly high [34]. For example, costs of advanced temperature monitoring automated response systems like RFID instead of the simple bar code system [22], along with costs associated with mandatory refrigerant changeover with upgrading efficiency and flexible capacity [23]. Zhu [45] reported that high investment costs had indeed been a major inhibitor in the diffusion of traceability systems in the food industry, in UAE. Operational costs have separately been considered as a significant measure of cold supply chains [46]. Ghadge et al [47] have included it as higher initial costs while establishing the barriers to the sustainable food supply chains in the context of UK. Rapid technological advancements also contribute to the hesitation of the decision makers, even though, it is up to a limited extent.

### Inadequate information system infrastructure

Due to the highly fragmented industry and a large number of players, insufficient IS infrastructure [34], along with information sharing add to the challenges. Resultantly, illegal, unreported, and unregulated (IUU) products also have a chance to enter the mainstream market. For food manufacturers seeking supply chain integration, Donk et al., [48] suggested that food supply chains should use concepts like EDI, VMI, QR, and CPFR for proper coordination of their supply chain practices. Konovalenko et al. [49] proposed a pioneer approach, which applies the Newton’s law of cooling (NLC) to volatile ambient temperatures. It uses a predefined set of conditions for temperature stability along with the component of error possible with the sensor measurement. Óskarsdóttir and Oddsson [50] developed a Decision Support Framework (DSF), which aims at helping the decision makers in identifying a feasible, sustainable and economical kind of traceability technology and structure for their products. A committed and resourceful team of decision makers is expected to get plenty of choices over next couple of years.

### Inadequate cold storage infrastructure

Lack of sufficient and efficient cold storage infrastructure [16] leads to carbon emissions from poorly controlled energy usage, leakage of refrigerant and emissions out of food waste [34] lead to adverse environmental effects [4]. Considering the maintenance of required temperature and humidity as an important determinant of product quality, Zhao et al. [51] proposed an innovative method for designing the cold supply chain design for ensuring an express delivery of strawberry fruits in the absence of the constantly ambient temperatures. While examining the performance and conduct of farmed oysters supply chains, Love et al. [52] considered the compliance with temperature regulations as one of the factors. The compliance to the desired temperature again demands the specific cold storage infrastructure suiting to the needs of varied assortments.

### Improper tracking

This may result in issues related to the food product being Halal [24], which in turn is related to food safety and food quality. For instance, the bottled water needs, among many, a halal certification by the competent federal authority before being commercialized. Thus, as a remedial measure, a number of researchers [53–56] have called for a reliable IT infrastructure for the cold supply chain. Aung and Chang [57] argued that efficient traceability systems would facilitate in minimizing production and distribution of unsafe or unsatisfactory quality products, which would result in a smaller number of reported cases for lousy publicity, liability, and recalls. Wu et al [58] also considered the proper tracking system as a prerequisite in striking a balanced trade-off between the quality of products in a cold supply chain and the energy requirement and emissions. Albrecht et al. [59] advocated the usage of Time Temperature Indicators (TTI) so that shelf life might be predicted in the light of microbial shelf life of the product.

### Lack of skilled labor

Skilled and trained staff is inadequate in terms of their familiarity with advanced equipment, and various IT systems. Industry-specific inventory management and handling practices and required specialized training [13] have been considered as a challenge thereof. Swift technological advancements require the skilled labor to be updated about the various operational aspects and the associated risks. Given the poorly skilled labor [34], cross docking, and a credible temperature assessment would affect the cold supply chain management particularly for fresh foods [36].

### Reliability issues with third-party logistics

It simply refers to dependability issues related to transportation and handling systems [3]. These include complete or partial failure to control the cold supply chain temperature during the transition, avoidance of simple administrative failures, such as not following shipment directions, or an unexpected regulatory inhibitor [60]. At times, the temperature abuse happens at the product receiving stage as well as the product dispatch stage, which has serious implications [61]. The trend of outsourcing has facilitated so many things, however, such issues have become multifold.

### Customers’ limited awareness about the quality dimensions

Experts also discussed at length, and negated other listed inhibitors like reverse logistics, poor road and transport, weak regulations, especially in the context of UAE. However, they suggested that customers’ limited awareness about the various dimensions of quality like expiry dates, required minimum temperature, humidity, lightning, etc. might have influenced the real goals of operating sustainable cold supply chains. Farooq et al., [40] acknowledged that the contamination caused by food degradation, food adulteration, counterfeiting or fake labelling etc. are some existing challenges in the food industry that affect consumer health and safety. In the context of barriers to sustainable food supply chains, Ghadge et al. [47] have referred to this dimension as the customers’ eco-literacy. The unavailability of arable land and local production of staple food items has deprived the local from being acquainted with the nuances of food items to certain extent.

Having extracted and discussed the most relevant inhibitors to the cold supply chain operations, lets delineate the research gap. Evidences in literature show that the researchers have studied different dimensions of the cold supply chain viz. enablers, barriers [4, 27], practices [20, 49], performance [45, 60], risk management [9, 54], competitiveness [15], cold chain network [39] etc. using different methodologies; (systematic) literature review [7, 41], bayesian approach [7], petri net modeling [9], ISM and MICMAC [17, 46], optimization approaches [23], system dynamic approach [36], and failure mode effect analysis [61]. Furthermore, the scope of these studies vary greatly; food cold supply chain [8, 47], perishable food supply chain quality [10], vaccine cold chain [13], sea-food supply chain, halal food supply chain [24], fruits & vegetables supply chain [25], processed food supply chain [26], pineapple supply chain [42], strawberry supply chain [51], dairy supply chain [56]. Moreover, these studies studying various dimensions of cold supply chains were conducted at different points of time and in different regional contexts; India [12, 16], Vietnam [27], UAE [33], Ghana [42], USA [52]. Technology is revolutionizing the whole industry as an incredible pace which is bringing up breakthrough success. Socio-cultural aspects as well as demographic dividends are also under transition. Overall, the business environment is become less and less predictable now. Hence, over time, the various factors (i.e. drivers, barriers, facilitators etc.) influencing a phenomenon also get changed. However, the pace of this transformation may be disproportionate among nations; underdeveloped world mostly remains underprivileged and lags behind. The salient features and uniqueness of this region (UAE) have already been elaborated at length at the end of the previous section. Furthermore, to the best of the researchers’ accessibility, not a single study was found to focus on the inhibitors encountered by the cold supply chains operating in Gulf Cooperation Council in General and United Arab Emirates (UAE) in particular. In fact, the studies in this research domain have been conducted in the countries which are blessed with the arable land, moderate climatic conditions, and agrarian societies etc. Hence, the output of this study is expected to fill this void and since, the other five nations of GCC region also share a similar socio-economic, natural, cultural and political profile, the trailblazer outcomes may be a stepping stone for a large-scale confirmatory study spanning across the GCC countries.

Based on this extensive literature review, the identified relevant inhibitors were used to draft a questionnaire, which was presented to ten experts: eight from the food industry and two from the academia. These seasoned individuals were requested to examine the listed inhibitors, add the omitted but otherwise significant ones in the context of this study, and eliminate the redundant ones. In this process, the initial list of 17 inhibitors was narrowed down to 12, which were used for ISM modeling.

## Objective and methodology

This section attempts to explain the methodological approach adopted to accomplish the broader objectives. It elaborates all the steps involved in carrying out this research.

### Objectives

This study attempts to: a) identify relevant inhibitors in the successful operations of cold supply chains in UAE b) explore and establish a structural relationship among these inhibitors and, c) classify the identified inhibitors into derived and independent inhibitors using FISM along with their dominance.

### Fuzzy interpretive structural modeling

Dealing with complex situations involving many variables has always been a herculean task, especially when they interact with each other. It tends to thwart any possible illustration (simplification) of the situation. A structural relationship among various elements pertaining to a system may be explored using ISM [62]. The expert opinions and judgments regarding the presence, degree, and direction of the relationship among these variables make this method interpretive; the depiction of specific relationships and overall structure on diagraph make this method a unique modeling technique.

Fuzzy sets theory was incorporated to further enhance the robustness of the proposed hierarchical model of the inhibitors as the basic ISM model is incapable to explain anything beyond the direction of relationship between and among the inhibitors. Fuzzy sets are relatively simple to administer than the other alternatives i.e. intuitionistic fuzzy values approach (IFVS) or neutrosophic sets or even rough sets. Being a generalization of the fuzzy sets, the IFVS takes the membership, non-membership as well as hesitation values in to consideration which demands relatively more rigour. As the selection of the experts was carefully done based on their academic and professional credentials, the researchers were confident about their thoroughness and non-hesitation. Hence, fuzzy set concept was deemed to suffice here. Fuzzy set theory is deemed capable of handling uncertainty efficiently, whereas neutrosophic set theory is considered efficient in tackling the indeterminate and inconsistent information [63]. Since the researchers were seeking the consensus among the experts, the elements of indeterminacy as well as inconsistency are automatically eliminated before a consensus is reached at. In fact, the intuitionistic or neutrosophic or rough sets are bring frequently used as complimentary concepts to the fuzzy sets in different context now a days. It is also quite noteworthy to mention here that the researchers couldn’t find a single similar study preferring to use intuitionistic fuzzy values approach, neutrosophic sets or even rough sets to examine the hierarchical relationship among the inhibitors of cold supply chain. Thus, in this case, the researchers preferred the fuzzy set theory over other theories of uncertainties and vagueness.

FISM is just another extension of ISM, which addresses the dominance of interaction related to the limitations of ISM [64]. The FISM process transforms unclear, poorly enunciated cognitive models of systems into vivid, well-defined models by employing a dominance of an interaction on a 0-l scale [64]. Having mentioned the dominance of interaction between and among the various sub-elements, FISM provides an interpretive framework of these sub-elements with one or two dominant interactions at a time, ignoring all other dominant interactions. For instance, if the researcher is interested in exploring only the high dominance of interaction, (s) he may retain only the entries of 0.7 and 0.9, and may replace all other entries to 0’s in the reachability matrix. In brief, the fuzzy interpretive structural modeling coupled with FMICMAC analysis is advantageous over the basic interpretive structural modeling in that it explains the strength of a specific relationship in addition to the direction of a relationship. Most of the research work on cold supply chain inhibitors as accessed and reviewed by the researchers so far have been carried out using ISM, FISM, or any MCDM or a combination of these. MCDM are usually applied to rank or classify certain elements pertaining to a phenomenon, whereas, the FISM-FMICMAC helps the researchers in exploring the interactive relationship between and among these elements on one hand and classify them into autonomous, independent, dependent and linkage elements on the other.

The FISM technique involves a number of steps [65], including the identification of elements through review of previous studies, which are then contextually validated by experts. Further, one needs to establish a contextual bearing among them, develop an initial self-interaction matrix for transitivity check, which would lead to the final reachability matrix. Post this convert the final reachability matrix in a fuzzy reachability matrix by adding the respective interaction dominance, partitioning the final reachability matrix, drawing a diagraph, and ultimately converting this diagraph into an FISM model. Nevertheless, this FISM model is checked for any likely conceptual inconsistency so that any necessary modifications may be made. Except the process flow diagram (illustrating the steps) and the fuzzy scheme illustration, researchers in this domain of research have not reported any mathematical formulation or equations in the Fuzzy ISM and Fuzzy MICMAC approach. Some of the recent research studies published based on this methodology in the high rank journals of their respective discipline include Sharma et al., [66], Das et al., [67], Singh et al., [68], Sharma et al., [69], Faisal et al., [70], and Melewar et al., [71].

Researchers in past selected and studied various aspects of cold supply chains using different methodological approaches viz. mathematical modeling in a simulated environment, multi-objective linear, non-linear or goal programming or the multi-criteria decision-making, and primary data-based analysis. Mathematical models are mostly proposed for the purpose of optimizing the various CSC flows. Furthermore, the other approaches like rough set theory or neutrosophic set theory, which are mostly used in computer science research particularly to deal with big data. The researchers in this research domain have been mostly using the fuzzy ISM to pursue one or more of the similar research objectives [67, 72]. However, the uniqueness of this study in terms of its context makes it imperative upon the researchers to explore the variables of interest, refine them with the help of experts and then examine any likely relationship among the final elements. Usually, when there are more than fifteen (15) variables related to any specific issue, the researchers try to reduce them using some factor reduction approach (like EFA). Thereafter, Interpretive Structural Model is applied. Some other researchers have also extended it further by using any multi-criteria decision-making tool Viz. Fuzzy AHP [73], ISM-FMICMAC-AHP-VIKOR [74], BWM [75], DEMATEL [76] etc. Ganguly and Das [73] have used ISM to examine the interaction between the barriers to stone crushing operations and Fuzzy AHP to further rank these barriers. Ray and Khaba [74] examined the ethical issues pertaining to green procurement in automobile industry using ISM-FMICMAC-AHP and ranked the various solutions using the VIKOR methodology. Amjadian et al., [75] developed a financial performance evaluation framework for the companies active in Tehran Stock Exchange. For this, the researchers finalized forty-nine financial ratios and consolidated them in to six broad categories. The structural relationship among these six categories of financial ratios was examined using ISM and MICMAC analysis whereas their ranking was done using BWM method. Shakeri and Khalilzadeh [76] used ISM to prioritize the factors affecting project communications and used DEMATEL to obtain the priority and magnitude of quantified relationships among these factors. There are plenty of such examples where the ISM method has been used in sync with some other quantitative and qualitative approaches.

In the present study, the researchers preferred FISM to explore a likely relationship among the selected inhibitors as it does not only reveal the existence of relationship or the direction of the relationship, but also provides a ground to determine whether a particular relationship emerged out of ISM process holds a merit to be considered significant for further decision making. This is done in the light of fuzzy score awarded by the researchers.

Agrawal et al., [77]; Behl et al., [78]; Gardas et al., [79]; Pramod and Banwet [80]; Bag [81]; and Abbas [82] have also applied the ISM approach for carrying out similar studies. In the context of the present study, these steps are elaborated. Three sectors, Meat & Fish (M&F), Milk & Milk Products (MMP), and Fruits & Vegetables (F&V) were chosen for this research, as the temperatures for M&F, MMP and F&V range (-180 C to +100 C) to cover most types of perishable items. Besides, the related supply chain crosses national and geographical boundaries, testing the mettle and potential of the available cold supply chain infrastructure to meet the larger market requirements.

Initially, the researchers visited four selected organizations to understand their cold supply chain integrity in managing the supply chain of perishable items. Post this visit, eight experts, two from each organization, holding the position of an assistant manager and above, along with two experts from academia who come from the operations and supply chain background, were approached for their valuable inputs. The number of expert opinions was determined judiciously in the light of previous studies, which were similar and engaged three to fifteen experts for such purposes [27, 35, 83, 84]. These experts were briefed about the subject of inquiry before being interviewed, using a semi-structured questionnaire. After a comprehensive examination of the experts, the researchers who had initially identified 17 inhibitors reduced them to 12. This initial screening of the inhibitors differs from Gligor et al., [27] who considered lack of government support for local businesses, social norms and absence of quality and safety-control initiatives at national level.

**Fig 1:**
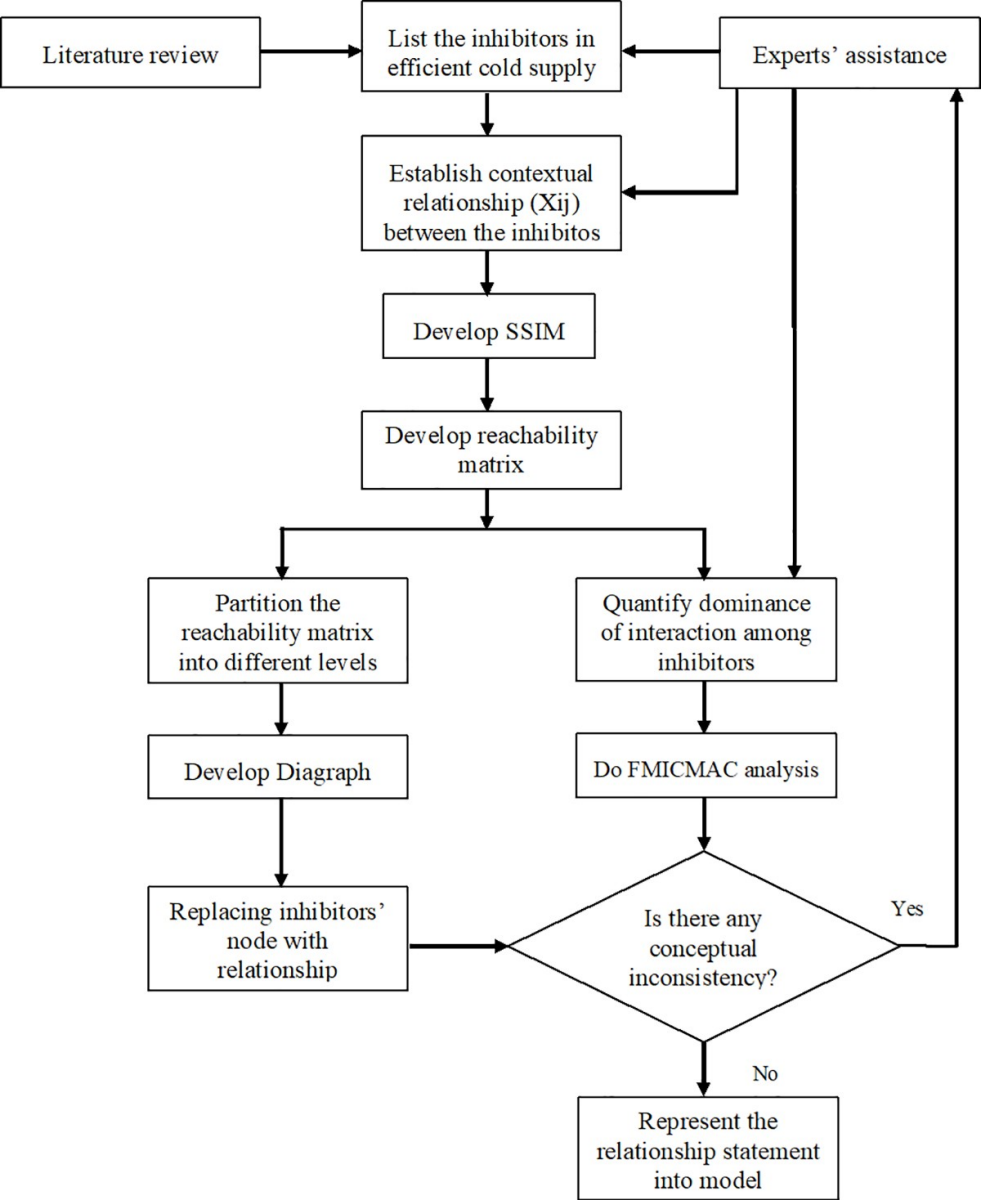
Flow diagram illustrating the FISM procedure (adopted from Joshi et al., [65]).

At the next stage, these experts were requested to establish any possible reciprocal relationships between pairs of variables (e.g., how a particular variable leads or supports other variables). The list of variables as identified, along with the structural self-interaction matrix (SSIM) of the elements developed on the basis of an agreement showing the reciprocal relationship among selected inhibitors has been circulated to experts for any possible modification. Finally, with their final agreement on those 12 variables, the FISM based model was developed. Fig 1 depicts the whole process.

Following the FISM technique [65], this study proceeds as follows;

**Step I:** The incorporation of the experts’ opinion into the findings of the review of relevant literature resulted in addition and elimination of some variables. The following table (Table 1) summarizes the list of inhibitors, which were finally considered for further investigation in present study.

**Table 1:** Selected inhibitors for the current study.

Sr.	Inhibitors	Brief description	
1	Fragmented cold supply chains (I_1_)	It involves many business partners (intermediaries, agents, etc.) from source (farm to fork) to destinations.	[3, 16, 25, 65, 81]
2	Lack of commitment by top-level management (I_2_)	This refers to the degree of reluctance vis a vis a strategically viable and operationally sustainable, which is exhibited in the attitude of top-level management.	[85, 94]
3	Poor cold supply chain network (I_3_)	It refers to those poorly designed networks, which fail to optimize the potentials of the network partners by integrating forward from suppliers to the consumers.	[86, 87]
4	Poor collaboration (I_4_)	It refers to the inability of management to properly coordinate, cooperate, and collaborate throughout the network to share forecasts, manage inventories, schedule labor, or optimize deliveries, etc.	[38, 88]
5	Negligible local production of selected CSC items (I_5_)	Being deprived of arable land, the local production of the selected cold supply chain items are minimal.	Due to the lack of arable land and the harsh environmental conditions, the local production of the selected items is negligible.
6	Higher capital & operating costs (I_6_)	Fixed costs (like importing, installing, and operationalizing the equipment) and operational costs (running and maintenance costs) are high.	[89, 90]
7	Inadequate information system infrastructure (I_7_)	Inadequate IS infrastructure here mostly concerns with the one at the suppliers’ end at source and during transit before these are delivered at their ultimate destination.	[91, 92]
8	Inadequate cold storage infrastructure (I_8_)	Inadequate infrastructure here mainly refers to the uneven infrastructure dealing with such supplies at source and during the transition before these are delivered at their ultimate destination.	[19, 93]
9	Improper tracking (I_9_)	Due to the fragmented nature of cold supply chains and inadequate IS infrastructure, tracing the product throughout the movement is challenging.	[52–54, 60]
10	Lack of skilled labor (I_10_)	Skilled and trained staff is inadequate in terms of their familiarity with modern equipment, IT systems, industry-specific stocking and handling practices, etc.	[13, 94]
11	Reliability issues with third-party logistics (I_11_)	In the absence of vertical integration, third-party logistics providers always face a degree of suspicion on account of trust and reliability regarding the maintenance of the prescribed conditions throughout the movement and storage, the unexpected delays, breakdowns, cross-contamination, etc.	[3, 60]
12	Customers’ limited awareness about the quality dimensions (I_12_)	It generally refers to the customers’ inability to scrutinize anything other than the expiry date before they buy any such product.	[95, 96]

**Step II:** A conceptual relationship was established between the elements by asking whether a particular inhibitor contributes towards the other inhibitor. This is done for all the inhibitors in a pairwise manner.

**Step III:** A structural self-interaction matrix (SSIM) of the elements (Table 2) has been established using the scheme, “**V** if inhibitor i contributes to inhibitor j; **A** if inhibitor j contributes to inhibitor i; **X** if inhibitors i and j contribute to each other; and **O** if neither of the two inhibitors i and j contribute to each other” [65], suggesting a pair-wise relationship between the system elements.

**Table 2:** Structural self-interaction matrix (SSIM).

	Inhibitors	12	11	10	9	8	7	6	5	4	3	2	1
**1**	Fragmented cold supply chains (I_1_)	O	O	A	V	X	A	O	A	O	V	O	-
**2**	Lack of skilled labor (I_2_)	V	V	A	V	V	X	V	A	O	V	-	
**3**	Poor cold supply chain network (I_3_)	O	V	O	V	A	O	V	O	V	-		
**4**	Poor collaboration (I_4_)	O	A	A	X	A	A	V	O	-			
**5**	Negligible local production of selected CSC items (I_5_)	O	O	X	O	O	O	V	-				
**6**	Higher capital and operating costs (I_6_)	O	A	A	A	A	A	-					
**7**	Inadequate information system infrastructure (I_7_)	V	O	A	V	O	-						
**8**	Inadequate cold storage infrastructure (I_8_)	A	V	A	V	-							
**9**	Improper tracking (I_9_)	O	X	A	-								
**10**	Lack of commitment by top level management (I_10_)	V	V	-									
**11**	Reliability issues with third-party logistics (I_11_) Customers’	O	-										
**12**	limited awareness about the quality dimensions (I_12_)	-											

**Step IV:** An initial reachability matrix (Table 3) has been constructed from the SSIM and the matrix is tested for transitivity in order to transform it into a final reachability matrix. This binary table is derived from the substitution of letters A, V, O, and X by 1 or 0 as specified in the following rule [62].

**Table 3:** Initial reachability matrix developed from the SSIM.

	Inhibitors	1	2	3	4	5	6	7	8	9	10	11	12
**1**	Fragmented cold supply chains (I_1_)	1	0	1	0	0	0	0	1	1	0	0	0
**2**	Lack of skilled labor (I_2_)	0	1	1	0	0	1	1	1	1	0	1	1
**3**	Poor cold supply chain network (I_3_)	0	0	1	1	0	1	0	0	1	0	1	0
**4**	Poor collaboration (I_4_)	0	0	0	1	0	1	0	0	1	0	0	0
**5**	Negligible local production of selected CSC items (I_5_)	1	1	0	0	1	1	0	0	0	1	0	0
**6**	Higher capital and operating costs (I_6_)	0	0	0	0	0	1	0	0	0	0	0	0
**7**	Inadequate information system infrastructure (I_7_)	1	1	0	1	0	1	1	0	1	0	0	1
**8**	Inadequate cold storage infrastructure (I_8_)	1	0	1	1	0	1	0	1	1	0	1	0
**9**	Improper tracking (I_9_)	0	0	0	1	0	1	0	0	1	0	1	0
**10**	Lack of commitment by top level management (I_10_)	1	1	0	1	1	1	1	1	1	1	1	1
**11**	Reliability issues with third-party logistics (I_11_)	0	0	0	1	0	1	0	0	1	0	1	0
**12**	Customers’ limited awareness about the quality dimensions (I_12_)	1	0	0	0	0	0	0	1	0	0	0	1

An entry of “V” in (I, j) cell of SSIM becomes 1 in the reachability matrix and the (j, i) entry becomes 0. An entry of “A” in (i, j) cell of SSIM becomes 0 in the reachability matrix, and the (j, i) entry becomes 1. An entry of “X” in (i, j) cell of SSIM becomes 1 in the reachability matrix, and the (j, i) entry also becomes 1. An entry of “O” in (i, j) cell of SSIM becomes 0 in the reachability matrix, and the (j, i) entry also becomes 0.

Thereafter, the initial reachability matrix is checked for the transitivity issues.

Transitivity rules holds that if “A” is related to “B” and “B” is related to “C”, then “A” is also related to “C”. After addressing the transitivity rule, the final reachability matrix (Table 4) is obtained.

**Table 4:** Final reachability matrix.

	Inhibitors	1	2	3	4	5	6	7	8	9	10	11	12
**1**	Fragmented cold supply chains (I_1_)	1	0	1	1*	0	1*	0	1	1	0	1*	0
**2**	Lack of skilled labor (I_2_)	0	1	1	1*	0	1	1	1	1	0	1	1
**3**	Poor cold supply chain network (I_3_)	0	0	1	1	0	1	0	0	1	0	1	0
**4**	Poor collaboration (I_4_)	0	0	0	1	0	1	0	0	1	0	1*	0
**5**	Negligible local production of selected CSC items (I_5_)	1	1	0	0	1	1	0	0	0	1	0	0
**6**	Higher capital and operating costs (I_6_)	0	0	0	0	0	1	0	0	0	0	0	0
**7**	Inadequate information system infrastructure (I_7_)	1	1	0	1	0	1	1	0	1	0	1*	1
**8**	Inadequate cold storage infrastructure (I_8_)	1	0	1	1	0	1	0	1	1	0	1	0
**9**	Improper tracking (I_9_)	0	0	0	1	0	1	0	0	1	0	1	0
**10**	Lack of commitment by top level management (I_10_)	1	1	0	1	1	1	1	1	1	1	1	1
**11**	Reliability issues with third-party logistics (I_11_)	0	0	0	1	0	1	0	0	1	0	1	0
**12**	Customers’ limited awareness about the quality dimensions (I_12_)	1	0	0	0	0	0	0	1	0	0	0	1

**Step V:** For the fuzzy process, the final reachability matrix is treated as binary direct reachability matrix (BDRM). Upon assigning the respective dominance of interactions (as per the scheme given in Table 5), the binary direct reachability matrix (BDRM) has been turned into a fuzzy direct reachability matrix (FDRM). It is done by giving the inhibitor a certain degree of perceived dominance factor following the below-mentioned scheme (adapted from [64]).

**Table 5:** Scheme for the degree of perceived dominance factor (adopted from [65]).

Dominance of interaction	No	Very low	Low	Medium	High	Very high	Full
**Grade**	N	NL	L	M	H	VH	F
**Value on the scale**	0	0.1	0.3	0.5	0.7	0.9	1

Generally, the driving power of each inhibitor is the total number of inhibitors (including itself) that it can lead to. On the other hand, dependence is the total number of inhibitors (including itself) that can contribute to it. In case of simple ISM technique that has crisp numbers (0, 1), the figures of driving and dependence is just obtained by counting the “1”. However, in FISM techniques where fuzzy values (continuous numbers between 0 and 1 in this case), the figures of driving and dependence is just obtained by adding the “non-zero” values. These driving powers and dependencies are subsequently used to classify inhibitors into four classes of autonomous, dependent, linkage and independent (driver) inhibitors (Table 6).

**Table 6:** Fuzzy direct reachability matrix (FDRM).

	Inhibitors	1	2	3	4	5	6	7	8	9	10	11	12	Driving Power
**1**	I_1_	1	0	0.9	0.7	0	0.5	0	0.9	0.9	0	0.7	0	**5.6**
**2**	I_2_	0	1	0.5	0.9	0	0.7	0.5	0.5	0.7	0	0.5	0.5	**5.8**
**3**	I_3_	0	0	1	0.9	0	0.7	0	0	0.9	0	0.9	0	**4.4**
**4**	I_4_	0	0	0	1	0	0.9	0	0	0.9	0	0.7	0	**3.5**
**5**	I_5_	0.9	0.7	0	0	1	0.3	0	0	0	0.3	0	0	**3.2**
**6**	I_6_	0	0	0	0	0	1	0	0	0	0	0	0	**1**
**7**	I_7_	0.7	0.7	0	0.9	0	0.7	1	0	0.9	0	0.9	1	**6.8**
**8**	I_8_	0.7	0	0.5	0.5	0	0.5	0	1	0.3	0	0.3	0	**3.8**
**9**	I_9_	0	0	0	0.9	0	0.7	0	0	1	0	0.9	0	**3.5**
**10**	I10	0.7	0.9	0	0.9	0.1	0.5	0.9	0.9	0.5	1	0.5	0.5	**7.4**
**11**	I11	0	0	0	0.9	0	0.5	0	0	0.1	0	1	0	**2.5**
**12**	I12	0.5	0	0	0	0	0	0	0.5	0	0	0	1	**2.0**
**Dependence**		**4.5**	**3.3**	**2.9**	**7.6**	**1.1**	**7.0**	**2.4**	**3.8**	**6.2**	**1.3**	**6.4**	**3.0**	

**Step VI:** The final reachability matrix was partitioned into different levels. Here, from the fuzzy direct reachability matrix (Table 6), the reachability set and antecedent set are obtained for each inhibitor. The reachability set comprises of an inhibitor itself along with others, which it may help to achieve, whereas the antecedent set comprises of the element itself along with other elements, which may help to achieve it. After finding these two sets, another set named intersection set is obtained based on the elements that are common in both the reachability and antecedent sets. The inhibitors, for which the reachability and intersection sets are the same, secure the top-level position in the FISM framework. Above their level, the top-level inhibitor is not going to help achieve any other inhibitor. Therefore, these inhibitors are eliminated for the next level of partitioning. In this manner, this process is continued until all inhibitors are leveled. Based on the various levels, one makes the diagraph and FISM model. In this case, Tables 7–13 show the reachability set, antecedent set, intersection set, and the various levels.

**Table 7:** Level partitioning iteration -1.

	Inhibitors	Reachability Set	Antecedent Set	Intersection Set	Level
**1**	**I**_1_	1, 3, 4, 6, 8, 9, 11	1, 5, 7, 8, 10,12	1, 8	
**2**	**I**_2_	2, 3, 4, 6, 7, 8, 9, 11,12	2, 7, 10	2, 7	
**3**	**I**_3_	3, 4, 6, 9, 11	1, 2, 3, 8	3	
**4**	**I**_4_	4, 6, 9, 11	1, 2, 3, 4, 7, 8, 9, 10, 11	4, 9, 11	
**5**	**I**_5_	1,2, 5, 6, 10	5, 10	5, 10	
**6**	**I**_6_	6	1, 2, 3, 4, 5, 6, 7, 8, 9, 10, 11	6	I
**7**	**I**_7_	1, 2, 4, 6, 7, 8, 9, 11, 12	2, 7, 10	2, 7	
**8**	**I**_8_	1, 3, 4, 6, 8, 9, 11	1, 2, 8, 10, 12	1, 8	
**9**	**I**_9_	4, 6, 9, 11	1, 2, 3, 4, 7, 8, 9, 10,11	4, 9, 11	
**10**	**I** 10	1, 2, 4, 5, 6, 7,8, 9, 10, 11, 12	5, 10	5, 10	
**11**	**I** 11	4, 6, 9, 11	1, 2, 3, 4, 7, 8, 9, 10, 11	4, 9, 11	
**12**	**I** 12	1, 8, 12	7, 10, 12	12	

**Table 8:** Level partitioning iteration -2.

	Inhibitors	Reachability Set	Antecedent Set	Intersection Set	Level
**1**	**I**_1_	1, 3, 4, 8, 9, 11	1, 5, 7, 8, 10,12	1, 8	
**2**	**I**_2_	2, 3, 4, 7, 8, 9, 11,12	2, 7, 10	2, 7	
**3**	**I**_3_	3, 4, 9, 11	1, 2, 3, 8	3	
**4**	**I**_4_	4, 9, 11	1, 2, 3, 4, 7, 8, 9, 10, 11	4, 9, 11	II
**5**	**I**_5_	1,2, 5, 10	5, 10	5, 10	
**7**	**I**_7_	1, 2, 4, 7, 8, 9, 11, 12	2, 7, 10	2, 7	
**8**	**I**_8_	1, 3, 4, 8, 9, 11	1, 2, 8, 10, 12	1, 8	
**9**	**I**_9_	4, 9, 11	1, 2, 3, 4, 7, 8, 9, 10,11	4, 9, 11	II
**10**	**I** 10	1, 2, 4, 5, 7,8, 9, 10, 11, 12	5, 10	5, 10	
**11**	**I** 11	4, 9, 11	1, 2, 3, 4, 7, 8, 9, 10, 11	4, 9, 11	II
**12**	**I** 12	1, 8, 12	7, 10, 12	12	

**Table 9:** Level partitioning iteration-3.

	Inhibitors	Reachability Set	Antecedent Set	Intersection Set	Level
**1**	**I**_1_	1, 3, 8	1, 5, 7, 8, 10,12	1, 8	
**2**	**I**_2_	2, 3, 7, 8,12	2, 7, 10	2, 7	
**3**	**I**_3_	3	1, 2, 3, 8	3	III
**5**	**I**_5_	1,2, 5, 10	5, 10	5, 10	
**7**	**I**_7_	1, 2, 7, 8, 12	2, 7, 10	2, 7	
**8**	**I**_8_	1, 3, 8	1, 2, 8, 10, 12	1, 8	
**10**	**I** 10	1, 2, 5, 7,8,10, 12	5, 10	5, 10	
**12**	**I** 12	1, 8, 12	7, 10, 12	12	

**Table 10:** Level partitioning iteration-4.

	Inhibitors	Reachability Set	Antecedent Set	Intersection Set	Level
**1**	**I**_1_	1, 8	1, 5, 7, 8, 10,12	1, 8	IV
**2**	**I**_2_	2, 7, 8, 12	2, 7, 10	2, 7	
**5**	**I**_5_	1,2, 5, 10	5, 10	5, 10	
**7**	**I**_7_	1, 2, 7, 8, 12	2, 7, 10	2, 7	
**8**	**I**_8_	1, 8	1, 2, 8, 10, 12	1, 8	IV
**10**	**I** 10	1, 2, 5, 7,8,10, 12	5, 10	5, 10	
**12**	**I** 12	1, 8, 12	7, 10, 12	12	

**Table 11:** Level partitioning iteration-5.

	Inhibitors	Reachability Set	Antecedent Set	Intersection Set	Level
**2**	**I**_2_	2, 7, 12	2, 7, 10	2, 7	
**5**	**I**_5_	2, 5, 10	5, 10	5, 10	
**7**	**I**_7_	2, 7, 12	2, 7, 10	2, 7	
**10**	**I** 10	2, 5, 7, 10, 12	5, 10	5, 10	
**12**	**I** 12	12	7, 10, 12	12	V

**Table 12:** Level partitioning iteration-6.

	**Inhibitors**	**Reachability Set**	**Antecedent Set**	**Intersection Set**	**Level**
**2**	**I**_2_	2, 7	2, 7, 10	2, 7	VI
**5**	**I**_5_	2, 5, 10	5, 10	5, 10	
**7**	**I**_7_	2, 7	2, 7	2, 7	VI
**10**	**I** 10	2, 5, 10,	5, 10	1, 2, 5, 10	

**Table 13:** Level partitioning iteration-7.

	Inhibitors	Reachability Set	Antecedent Set	Intersection Set	Level
**5**	**I**_5_	5, 10	5, 10	5, 10	VII
**10**	**I** 10	5, 10	5, 10	5, 10	VII

**Fig 2:**
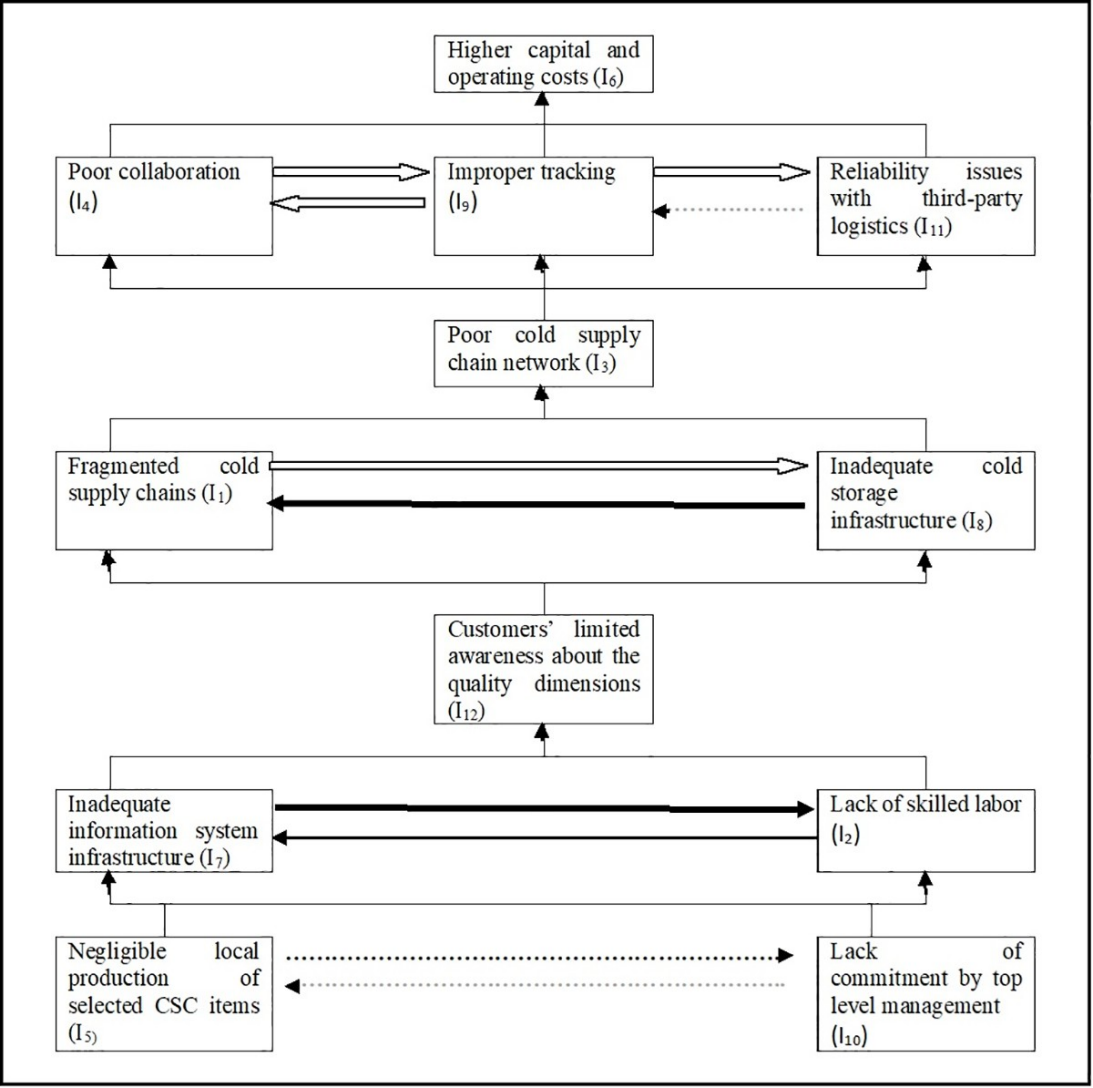
The FISM based model illustrating the structural relations with their respective dominance of interaction.

**Fig 3:**
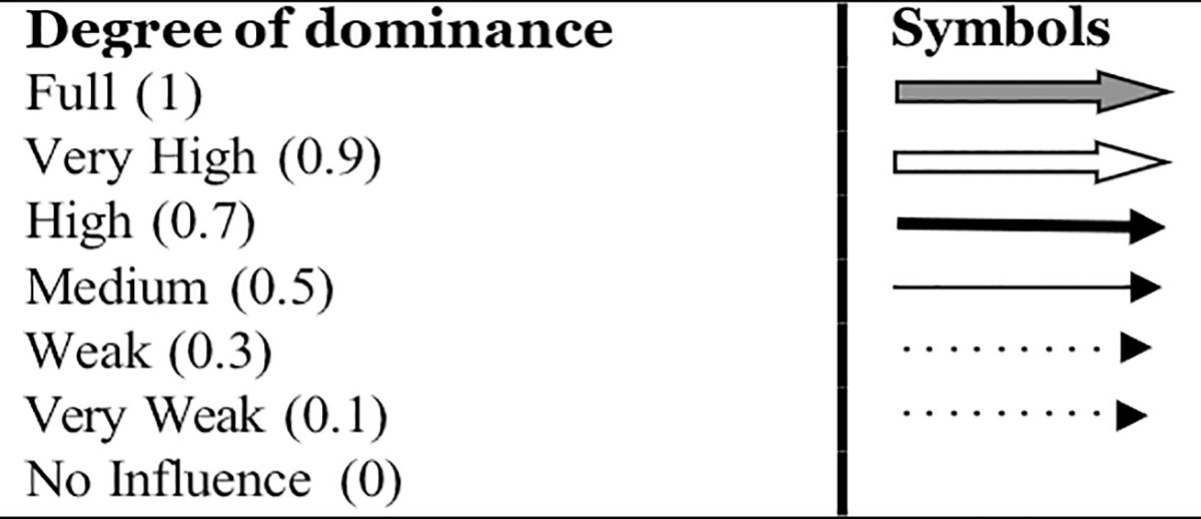
Notations.

**Step VII**: Considering the relationships that emerged from Tables 7–13, a directed graph (DIAGRAPH) is drawn, eliminating the transitive links. Furthermore, the resulting digraph is translated into a FISM by replacing element nodes with statements, and the dominance interactions. Though an analyst is free to study and consider any degree of dominance [65], the researchers have depicted full, very high, high, medium, weak and very weak degree of dominance. Hence, in the final FISM model (Fig 2), an arrow between any two inhibitors i and j, pointing from i towards j, shows that the inhibitor i derives inhibitor j, while the thickness of this arrow (as per Fig 3) shows the level of dominance of interaction. The inhibitors at the bottom-most level in this model have a ‘lead to’ kind of relationship with the dependent variables at the next level in the hierarchy.

**Fig 4:**
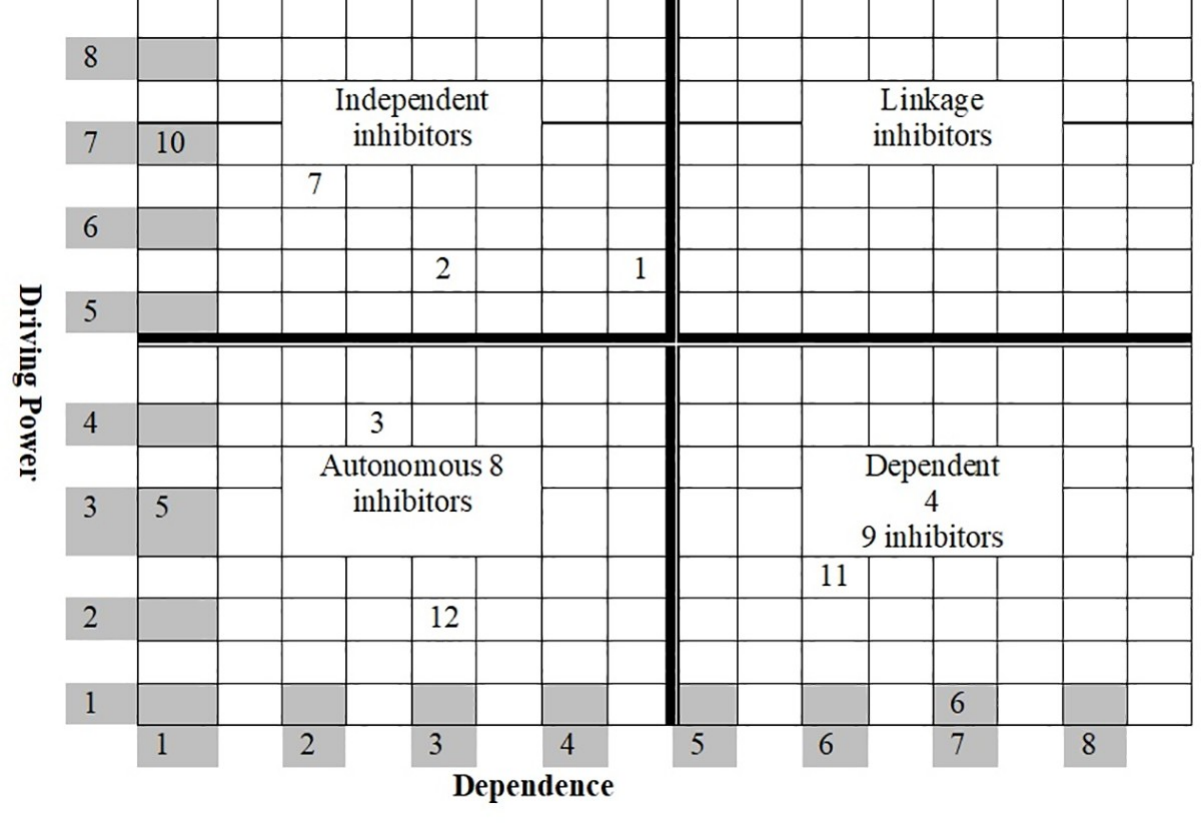
Driving power and dependence diagram (FMICMAC).

**Step VIII**: Taking into consideration the dominance of interaction, the Fuzzy MICMAC analysis identifies and analyses the inhibitors based on their driving and dependence power. This two-dimensional analysis helps the researchers categorizing various inhibitors into four different groups, as illustrated below in Fig 4. Except the third quadrant (linkage inhibitors), all of the remaining three quadrants receive some of the studied inhibitors.

The inhibitors, which have weak driving power and weak dependence, are classified as ‘autonomous inhibitors.’ Being plotted in the first quadrant, these are regarded as being relatively disconnected from the system because of having fewer or no strong links. The second quadrant contains the ‘dependent inhibitors,’ which have weak driving power, but strong dependence. The third quadrant has the ‘linkage inhibitors’ that have strong driving power and dependence. Being unstable inhibitors [82], any action on these would have an effect on the others, and consequently, a reflexive effect on themselves. The fourth quadrant consists of ‘independent inhibitors’ with strong driving power and weak dependence. The management should be extra cautious in dealing with these inhibitors, as they influence the impact of other variables appearing at the top of the ISM hierarchy in the overall cold supply chain management.

**Step IX**: The FISM model is reviewed to check for conceptual inconsistency, and the necessary modifications are made with the help of experts.

## Discussion, conclusion, implications and direction for future research

The current research has attempted to incorporate the degree of dominance of interaction, while modeling the structural relationship among various inhibitors of cold supply chains in the context of UAE. The proposed FISM model shows that ‘lack of commitment of the top management’ and ‘negligible local production of selected CSC items’ are occupying the lowest level in the hierarchy. In a similar study of modelling of supply chain management enablers using ISM and Fuzzy MICMAC analysis, Merwe et. al, [97] found the commitment of the top management as an enabler occupying the lowest level in the hierarchy.

Furthermore, the degree of dominance of interaction of ‘the lack of commitment of the top management’ with ‘inadequate information system infrastructure’ is very high. However, despite being at the same level, the degree of dominance of interaction of ‘the lack of commitment of the top management’ with ‘the negligible local production of selected CSC items’ seems to be either very weak or near to non-existent; same is the case with ‘lack of skilled labor’ at the next level in hierarchy. It is quite reasonable to conceive that availability of good quality local produce is going to contribute to the commitment of top management, though to a very limited extent. Likewise, a committed top management may endeavor to utilize the limited local resources within the policy framework of the local governance. However, the cost and operational concerns (like scale) may discourage them to do so. The ‘lack of commitment by the top management’ based on its driving power is considered to be a strong inhibitor, which generally tends to be the root cause of all the related inhibitors. As these results require the utmost attention of companies along with their management, the top management is expected to be more attentive, responsive, involved, and proactive to curb other related issues. Their enhanced engagement and commitment are directly going to reflect in a strong information system infrastructure, which may further help eliminate other inhibitors in the next levels of the hierarchy.

Generally, it has been observed that apart from the brand name, price, quantity, and date of expiry, a customer is not much bothered about the rest of the quality dimensions of the daily used products. Lack of proper information systems, along with the poorly skilled labor force, may further suppress the customers’ interest to know more. On the other hand, if there are skilled, qualified, and experienced human resources, equipped with advanced information system infrastructure, it may help manage the inventories better, and educate customers, which in turn would possibly result in raising the consumption and disposal standards of the common masses.

Sellers caring as much as buyers is also not an uncommon tendency; consequently, the limited awareness of customers about various quality dimensions discourages the companies (specifically the smaller ones) to heavily invest in creating the cold supply chain infrastructure beyond a minimum required level. The fragmented nature of the cold supply chains, which share the third level in this hierarchy with the inadequate cold chain infrastructure, further aggravates the reluctance towards investing heavily in it. Consequently, these two inhibitors lead to the worsening of the cold supply chain network, which occupies the second level in this hierarchy.

In the end, the resultant poor cold supply chain network leads to a multitude of issues like ‘poor collaboration,’ ‘improper tracking ‘and ‘reliability issues with third-party logistics’. Its dominance of interaction is also high with all these listed inhibitors, and even higher with the ‘reliability issues with third-party logistics.’

Further, it is noteworthy to mention that there is a high bi-directional dominance of interaction between ‘poor collaboration’ and ‘improper tracking.’ ‘Improper tracking,’ in its turn, has a high degree of dominance of interaction with ‘reliability issues with third-party logistics.’ Thus, logically, one could say that if the supply chain network were weak, it would be difficult for business partners to coordinate and collaborate their business activities.

Additionally, it will be difficult to trace what happened when. All this in amalgamation would lead the entire system to lose its reliability with stakeholders. Thus, a weak supply chain network derives the poor collaboration, poor tracking and diminishing reliability ultimately culminates into a burden of increased fixed as well as variable costs. Hence, higher fixed and variable costs occupy the top most level in the FISM hierarchy. Therefore, to sum up, it may be stated that companies represented by their top management must understand how the domino effect behind every single change they plan and bring about, as various inhibitors are inter-related in terms of structure and dominance of interaction.

Based on their driving and dependence powers, the fuzzy MICMAC analysis categorized these inhibitors into four different groups. The ‘poor cold supply chain network’, ‘negligible local production of selected CSC items’, ‘inadequate cold storage infrastructure’, and ‘customers’ limited awareness about the quality dimensions’ are grouped as autonomous inhibitors. These inhibitors have relatively a weak driving power and dependence; thereby they do not have much influence on the system.

Apparently, ‘negligible local production of selected CSC items’ seems to be a disconnected inhibitor at the lowest level in the FISM model. Nevertheless, the other three inhibitors viz. ‘poor cold supply chain network’, ‘inadequate cold storage infrastructure’, and ‘customers’ limited awareness about the quality dimensions’, constitute the other higher levels of hierarchy. One possible reason could be due to their relatively moderate level of driving or dependence power. The management may focus on such inhibitors when it looks to address the other, more influential inhibitors.

The ‘poor collaboration’, ‘higher capital and operating costs’, ‘improper tracking’ and ‘reliability issues with third-party logistics’ have been classified as dependent inhibitors. These are weak in terms of their driving power, but are strong in terms of their dependence power. The management should thereby examine to which lower-level inhibitors, these depend on; and begin to address them accordingly. Despite sharing the same levels, no pair of the inhibitors could emerge to be the linkage inhibitors, due to rather moderate to weak driving power and dependence. For instance, at the lowest (VII*th*) level of the FISM model, the ‘negligible local production of selected CSC items’ and ‘lack of commitment by top-level management’ lie. However, due to their poor degree of dominance of interaction between them, they do not appear in the category of linkage inhibitors. As the ‘higher capital and operating costs’ is the top-level inhibitor in the hierarchy, it cannot derive any inhibitor below its own level in the hierarchy.

Even technically, a higher-order inhibitor cannot derive a lower order inhibitor. The ‘fragmented cold supply chains’, ‘lack of skilled labor’, ‘inadequate information system infrastructure’ and ‘lack of commitment by top level management’ are classified as independent inhibitors. They have a strong driving power, but low dependence power; hence, these are mostly considered as the most significant inhibitors. In most of the cases, these come out as being the root cause of many other related inhibitors.

Therefore, the management must not be oblivious to such inhibitors; in fact, managerial in attention to these inhibitors could create many unprecedented situations.

The findings of present study differ from Bag [81] who used ISM methodology to examine ten inhibitors of green cold chain management as the inhibitors ‘poor cold chain network’, ‘lack of information system infrastructure’ and ‘outdated technology and poor cold storage infrastructure’ occupy third, fourth and fifth levels respectively. This study also differs from Joshi et al., [65] in terms of the specific inhibitors examined for cold supply chains. The lack of awareness about the use of IT, absence of quality and safety measures, inadequate farmers’ education, lack of standardization, government regulation were found contextually less relevant or irrelevant at all; hence, these were not considered. This study is also different from Shashi et al., [8] in terms of the selected cold chain inhibitors, where some less relevant or irrelevant inhibitors like the lack of specific environmental goals, unavailability of cold chain performance measurements, uneven installation of cold chain centers, lack of awareness about adopting reverse logistics and lack of government support, etc., were not at all considered for further examination. In fact, the findings also differ; for instance, the inadequate cold chain infrastructure, high investment costs, high-energy costs had been identified as the first three significant inhibitors for cold supply chains; whereas, in the current study, these represent negligible local production of selected CSC items, along with the lack of commitment by the top management. However, the findings of this study corroborate the outcomes of another study conducted by Tamimi et al., [33] about cold chain logistics implementation strategies in the context of UAE. More specifically, the cost concerns and constant monitoring along with control mechanism related to the cold supply chain implementation have been highlighted in the current study as well. Furthermore, it is also culminating up to higher fixed and operational costs which is similar to findings of Al-Refaie et al. [46]. The present model, along with the issue of the lack of skilled staff, is also in line with Sundarakani et al., [98] who noted that the UAE-based companies are required to ‘educate’ their managerial staff about the plethora of IT systems benefits like reduction in cycle time and improvement in supply chain visibility. Being primarily used for transactional processing, the utility of IT systems may be extended to the planning and better decision-making domains.

To sum up, it can be stated that while corroborating with some similar studies carried out from different perspectives in different context, this study also distinguishes itself from some others. The identification and establishment of the key inhibitors impeding the CSC operations in UAE and the portrayal of their evidence-based mutual relationships in the form of an easy to understand FISM model is amount to the major contribution of this study. Furthermore, the classification of these inhibitors in to different categories viz. dependent, independent, and autonomous and linkage categories is another significant add-on.

Despite its usefulness and value addition in cold supply chain literature, this study as well as methodological approach has certain limitations. Being limited to the review of literature and judgements of experts makes the identification of relevant inhibitors vulnerable to issue of exhaustiveness. Furthermore, the set of inhibitors to the cold supply chain may also vary as the geographic region changes. For instance, negligible local production of CSC items due to unavailability of arable land which is considered a significant inhibitor in case of GCC region may become a completely insignificant inhibitor in Indian subcontinent. Hence, the study findings may not be generalized. Besides, the number of cold supply chain items, if increased (by adding other cold supply chain items like pharmaceuticals, flowers etc.), a different set of inhibitors may emerge. Thus, the outcome of this study may not be related to all kinds of cold supply chains. In addition to the limitations pertaining to the number of representative organizations, number of respondents, disparities in their knowledge, experience and overall educational, professional, socio-cultural backgrounds, certain methodological limitations also exist. For instance, subjectivities involved in the selection of the representative firms and the representative experts, the decision about the exhaustiveness of responses, the individual judgements of experts, their willingness, capability and degree of engagement, the issues related to group conformance in order to reach a consensus, or inability to model uncertainties in case of incomplete information make the research output open to another confirmatory study. The future researchers may use different objectives, different methodological approaches like any multi-criterial decision analysis tools (e.g. DEMATEL for further classifying these inhibitors) or blended toolkit like mixed method approach, expand the topical coverage, increase the number of participating firms, involve more number of experts, involve respondents from other GCC countries etc. to further obtain the additional insights. The future researchers may work upon these limitations, carry out sensitivity analysis and further examine the broader implications and generalizability of these outcomes.

## Supporting information

S1 File Data-sheet. (DOCX)
