# Supplementary material for: Modelling the inhibitors of cold supply chain using fuzzy interpretive structural modeling and fuzzy MICMAC analysis
Source: PLoS One. 2021 Apr 13;16(4):e0249046. doi: 10.1371/journal.pone.0249046 (PMC8043388; doi:10.1371/journal.pone.0249046)
Supplement: S1 File — (DOCX) [file pone.0249046.s001.docx]

**Data-Sheet**

**Q1. Use the below mentioned matrix to develop a Structural Self-Interactive Matrix (SSIM) based on understood contextual relationships following the following letter scheme.**

Fill V if Inhibitor i will leads to inhibitor j; A if Inhibitor j leads to inhibitor i; X if Inhibitors i and j lead to each other; and O if Inhibitors i and j are unrelated.

|  |  | 12 | 11 | 10 | 9 | 8 | 7 | 6 | 5 | 4 | 3 | 2 | 1 |
| --- | --- | --- | --- | --- | --- | --- | --- | --- | --- | --- | --- | --- | --- |
| 1 | Fragmented cold supply chains | O | X | O | X | A | A | X | O | V | V | O |  |
| 2 | Lack of skilled labor | V | X | V | V | X | X | X | O | V | V |  |  |
| 3 | Poor cold supply chain network | O | A | A | A | A | A | A | O | V |  |  |  |
| 4 | Poor Collaboration | X | A | X | A | A | A | A | O |  |  |  |  |
| 5 | Negligible Local production of selected CSC items | O | O | A | O | O | O | A |  |  |  |  |  |
| 6 | Higher Capital and Operating Costs | V | V | V | V | X | X |  |  |  |  |  |  |
| 7 | Inadequate Information System Infrastructure | V | V | O | V | O |  |  |  |  |  |  |  |
| 8 | Inadequate cold storage infrastructure | O | V | O | V |  |  |  |  |  |  |  |  |
| 9 | Improper Tracking | V | V | A |  |  |  |  |  |  |  |  |  |
| 10 | Lack of commitment by top level management | V | V |  |  |  |  |  |  |  |  |  |  |
| 11 | Reliability issues with third-party logistics | O |  |  |  |  |  |  |  |  |  |  |  |
| 12 | Customers’ limited awareness about the quality dimensions |  |  |  |  |  |  |  |  |  |  |  |  |

**Q2. Follow the below mentioned scheme [Adapted from Saxena et al. (2006)] to express your perceived dominance of the interaction between a pair of inhibitors.**

| **Dominance of interaction** | No | Very low | Low | Medium | High | Very high | Full |
| --- | --- | --- | --- | --- | --- | --- | --- |
| **Grade** | N | NL | L | M | H | VH | F |
| **Value on the scale** | 0 | 0.1 | 0.3 | 0.5 | 0.7 | 0.9 | 1 |

|  |  | 12 | 11 | 10 | 9 | 8 | 7 | 6 | 5 | 4 | 3 | 2 | 1 |
| --- | --- | --- | --- | --- | --- | --- | --- | --- | --- | --- | --- | --- | --- |
| 1 | Fragmented cold supply chains | L | H | M | VH | H | H | H | N | VH | VH | N |  |
| 2 | Lack of skilled labor | H | H | H | H | H | H | M | N | F | F |  |  |
| 3 | Poor cold supply chain network | N | VH | H | H | H | H | H | N | VH |  |  |  |
| 4 | Poor Collaboration | N | VH | M | H | H | VH | H | N |  |  |  |  |
| 5 | Negligible Local production of selected CSC items | N | N | H | N | N | N | H |  |  |  |  |  |
| 6 | Higher Capital and Operating Costs | M | H | H | VH | VH | VH |  |  |  |  |  |  |
| 7 | Inadequate Information System Infrastructure | VH | F | N | VH | H |  |  |  |  |  |  |  |
| 8 | Inadequate cold storage infrastructure | N | VH | N | VH |  |  |  |  |  |  |  |  |
| 9 | Improper Tracking | M | VH | H |  |  |  |  |  |  |  |  |  |
| 10 | Lack of commitment by top level management | H | H |  |  |  |  |  |  |  |  |  |  |
| 11 | Reliability issues with third-party logistics | N |  |  |  |  |  |  |  |  |  |  |  |
| 12 | Customers’ limited awareness about the quality dimensions |  |  |  |  |  |  |  |  |  |  |  |  |
